# Adolescent Fentanyl Use: Toxicological Risks, Epidemiological Trends, and Public Health Strategies

**DOI:** 10.7759/cureus.79558

**Published:** 2025-02-24

**Authors:** Matteo Antonio Sacco, Saverio Gualtieri, Agostinho Santos, Bárbara Mendes, Isabella Aquila

**Affiliations:** 1 Department of Medical and Surgical Sciences, Magna Graecia University, Catanzaro, ITA; 2 Department of Legal Medicine and Forensic Sciences, Faculty of Medicine, University of Porto, Porto, PRT; 3 Department of Forensic Medicine, Instituto Nacional de Medicina Legal e Ciencias Forenses, Porto, PRT; 4 Department of Medical and Surgical Sciences, Institute of Legal Medicine, Magna Graecia University, Catanzaro, ITA

**Keywords:** adolescent, drug abuse, fentanyl, illicit drug, young adult behavior

## Abstract

Fentanyl has become a critical public health concern due to its increasing presence in illicit drug markets and its role in the rising number of opioid-related fatalities, particularly among adolescents. This study examines the toxicological properties of fentanyl, its accessibility through online platforms, and the behavioral factors contributing to its misuse in adolescent populations. Key findings highlight the high risk of overdose due to fentanyl’s potency, especially when combined with substances such as alcohol and benzodiazepines. Epidemiological data reveal a dramatic surge in adolescent fentanyl-related deaths, exacerbated by counterfeit prescription drugs and a lack of awareness regarding its extreme potency. The study also explores prevention and intervention strategies, including public health education, harm reduction approaches, and policy recommendations aimed at mitigating the growing crisis. Addressing this issue requires a multifaceted approach, integrating medical, social, and legislative efforts to protect vulnerable adolescent populations.

## Introduction and background

Fentanyl (N-phenyl-N-[1-(2-phenylethyl)piperidin-4-yl]propenamide) is a synthetic opioid with a molecular mass of 336.479 g/mol and a chemical structure that allows it to bind rapidly and effectively to μ-opioid receptors [1]. With an analgesic potency 50 to 100 times greater than morphine, fentanyl was originally synthesized by Paul Janssen in 1960 for use in anesthesia and the treatment of chronic pain. Its high lipophilicity (logP 2.3 at pH 7.4) enables it to cross the blood-brain barrier almost instantaneously, leading to rapid and profound analgesic effects [2]. Although fentanyl remains an essential drug in clinical medicine, particularly for palliative care and cancer-related pain management, its pharmacokinetic properties have made it a dangerous and highly sought-after substance in the illegal drug trade.

The pharmacodynamics of fentanyl involve binding to μ-, κ-, and δ-opioid receptors in the central nervous system (CNS), inhibiting the release of neurotransmitters such as glutamate and substance P [2]. This results in reduced neuronal excitability and a profound suppression of pain signals. However, the same mechanism also affects the brainstem's respiratory centers, reducing the body's sensitivity to carbon dioxide (CO₂) and causing respiratory depression, which is the leading cause of fentanyl-related deaths. This depressive effect on the respiratory system is further exacerbated when fentanyl is used in combination with other CNS depressants such as alcohol, benzodiazepines, and barbiturates, significantly increasing the risk of fatal overdose.

Historically, fentanyl was primarily used in controlled clinical settings. However, in recent years, it has found its way into the illicit drug market, often sold as counterfeit pills or mixed with other substances to enhance potency and profitability. The rise of cryptomarkets on the dark web has facilitated this distribution, providing anonymity to buyers and sellers alike. These markets allow users to purchase fentanyl and its analogs with cryptocurrencies such as Bitcoin, making the substance easily accessible to young and inexperienced users. Adolescents, driven by risk-taking behavior and a desire for novel experiences, are particularly vulnerable to the dangers of synthetic opioids, often underestimating their potency and the associated risks.

## Review

Materials and methods

A comprehensive literature review was conducted using the PubMed database, focusing on articles published between 2010 and 2023 to ensure the inclusion of the most current data. The keywords used for the search were (fentanyl) AND (adolescence) AND (overdose), combined with Boolean operators to refine the results. The review included original research articles, cohort studies, case reports, and epidemiological analyses that examined the toxicological effects of fentanyl and its analogs in adolescent populations.

Studies were selected based on their relevance to fentanyl use among adolescents aged 13-19 years, particularly those addressing the behavioral, clinical, and toxicological aspects of overdose cases. Priority was given to studies reporting on the co-administration of fentanyl with other psychoactive substances such as benzodiazepines, methamphetamine, alcohol, and xylazine, given the significant role of polydrug use in the rising mortality rates. The methodological approach also involved consulting epidemiological data from the Centers for Disease Control and Prevention (CDC), the European Monitoring Centre for Drugs and Drug Addiction (EMCDDA), and the World Health Organization (WHO) to contextualize the prevalence and impact of fentanyl-related deaths among adolescents.

Additionally, we assessed the risk of bias in included studies using standardized criteria, such as sample size representativeness, data collection methods, and potential conflicts of interest. Any discrepancies in study selection were resolved through discussion among the authors to ensure objective and comprehensive inclusion of relevant research. Despite these efforts, certain limitations must be acknowledged. Variations in study design and data collection methodologies among different sources may introduce inconsistencies in results interpretation. Addressing these limitations, future research should incorporate meta-analytical techniques and standardized reporting guidelines to enhance reproducibility in fentanyl-related studies.

Results and discussion

Epidemiological Trends in Adolescent Fentanyl Use

Adolescence is a critical developmental period marked by significant biological, psychological, and social changes [3]. During this stage, the adolescent brain undergoes extensive remodeling, particularly in areas associated with decision-making, impulse control, and emotional regulation. The prefrontal cortex - the region responsible for executive functions such as planning and risk assessment - develops more slowly than the limbic system, which is involved in reward processing and emotional responses. This neurodevelopmental imbalance predisposes adolescents to risk-taking behaviors, including substance use.

Fentanyl use among adolescents has seen a dramatic increase in recent years [3-4]. Epidemiological data from the CDC reveal that opioid-related overdose deaths involving fentanyl rose from 29% in 2015 to 82% in 2020 among adolescents aged 15-19 years [3-4]. The EMCDDA reports a similar trend in Europe, where fentanyl and its analogs have been responsible for a growing number of fatal overdoses. The increasing availability of fentanyl on the dark web, coupled with its low cost and high potency, has made it a particularly attractive option for young users [5].

One of the most concerning aspects of fentanyl use in this age group is the high rate of polydrug use. Adolescents often consume fentanyl alongside other substances such as alcohol, benzodiazepines, and stimulants, which exponentially increases the risk of overdose. These combinations are not only more dangerous but also more difficult to manage clinically, as the symptoms of intoxication and withdrawal can vary significantly depending on the substances involved. The metabolic breakdown of fentanyl through the cytochrome P450 system, particularly via the CYP3A4 enzyme, produces active metabolites that can prolong its effects and complicate treatment protocols [6].

Social and Psychological Consequences of Fentanyl Use

The social consequences of adolescent fentanyl use are equally severe. Substance use during adolescence can disrupt academic achievement, increase the likelihood of legal problems, and contribute to mental health disorders such as anxiety and depression. Moreover, the onset of substance use at an early age can have lasting effects on brain development, impairing cognitive function and emotional regulation well into adulthood [7].

Epidemiological data show an alarming increase in the prevalence of fentanyl-related morbidity and mortality among adolescents. Between 2013 and 2018, fentanyl overdose deaths in the United States rose tenfold, from approximately 3,000 to over 30,000 annually [8]. By 2021, fentanyl had become the leading cause of death among adolescents aged 10-19 years, surpassing fatalities from firearms and motor vehicle accidents. The CDC highlights that more than 1,100 adolescents aged 10-19 years died from fentanyl-related overdoses in 2021 alone. Non-fatal overdose events are equally concerning. Emergency department data from 2020 indicate that over 218,000 adolescents aged 15-24 years sought medical care for involuntary opioid poisoning, reflecting the widespread impact of fentanyl use in this age group [9-10].

In addition to mortality, the rise of non-fatal overdoses presents a significant burden on healthcare systems and families. Survivors of overdose often experience long-term neurological damage due to hypoxia caused by respiratory depression. These individuals may suffer from cognitive impairments, memory loss, and emotional dysregulation, which further complicate their reintegration into educational and social environments. The increased rate of exposure to fentanyl and other synthetic opioids highlights the urgent need for public health interventions tailored to the adolescent population [10].

Social and economic factors play a crucial role in the spread of fentanyl use among adolescents. Studies indicate that fentanyl misuse is disproportionately prevalent in low-income communities, where access to mental health services and educational resources is limited. Socioeconomic disparities exacerbate vulnerabilities, leaving adolescents in these environments with fewer protective factors and greater exposure to risk. Peer influence and the desire to achieve social acceptance further contribute to substance experimentation, particularly in environments where substance use is normalized.

The Role of Counterfeit Prescription Drugs

One of the most disturbing trends is the intentional marketing of counterfeit prescription drugs containing fentanyl. Many adolescents begin experimenting with substances under the misconception that they are taking a legitimate pharmaceutical product, such as oxycodone or alprazolam. These counterfeit pills, often purchased on social media platforms or the dark web, are visually indistinguishable from the real medication but contain lethal doses of fentanyl. This deceptive practice has been directly linked to the spike in adolescent overdose deaths in recent years.

The global opioid crisis is not limited to Western countries; it is also deeply influenced by drug production and trafficking networks in regions such as the Golden Triangle and Golden Quadrangle. These areas, historically known for heroin production, have increasingly become hubs for synthetic opioid manufacturing and distribution. The porous borders, weak regulatory frameworks, and presence of organized crime syndicates facilitate the production and export of fentanyl analogs, which often enter global markets through illicit supply chains.

Fentanyl's accessibility is further exacerbated by its low production costs and ease of transportation. Unlike heroin, which requires large-scale poppy cultivation, fentanyl can be synthesized in small clandestine laboratories with readily available precursor chemicals. This has made it a preferred choice for drug traffickers, enabling large-scale distribution via dark web marketplaces, postal services, and underground networks. The increasing integration of fentanyl into the global opioid supply raises significant public health concerns, as users often consume these substances unknowingly in counterfeit prescription pills or mixed with other drugs. Addressing the accessibility of fentanyl requires stronger international cooperation, stricter regulation of precursor chemicals, and more robust surveillance of online drug markets.

Prevention and Intervention Strategies

Given the complex nature of fentanyl addiction in adolescents, a multifaceted approach is essential for effective prevention and intervention. Public health strategies must prioritize education, early intervention, and community-based support systems. Schools should play a central role in raising awareness about the dangers of synthetic opioids. Comprehensive drug education programs should go beyond traditional messaging to include practical information on recognizing the signs of overdose, understanding the risks of counterfeit pills, and promoting the use of naloxone (Narcan) as a life-saving intervention [11-12].

Naloxone, an opioid antagonist that rapidly reverses the effects of opioid overdose, should be more widely available in schools, community centers, and other public spaces. Pediatricians and primary care providers must be proactive in discussing substance use with adolescents, screening for early signs of misuse, and educating families about the risks associated with opioids. Harm reduction strategies, such as needle exchange programs and supervised consumption sites, can also reduce the risks associated with fentanyl use in more vulnerable populations.

The role of social media in facilitating drug distribution and glamorizing substance use cannot be ignored. Public health campaigns must collaborate with social media platforms to monitor and restrict the sale of illicit substances online while promoting awareness initiatives that resonate with adolescents. Anonymous reporting tools and digital interventions, such as mobile apps that offer real-time information and support, could help reach at-risk youth more effectively [13-14].

Community engagement is critical in addressing the social determinants of substance use. Programs that provide mentorship, recreational activities, and academic support can reduce the likelihood of substance experimentation by offering adolescents alternative sources of fulfillment and connection. Family-based interventions, which focus on improving communication, building resilience, and addressing underlying mental health issues, are particularly effective in reducing substance use among adolescents.

On a policy level, stricter regulations on the distribution of synthetic opioids and increased funding for mental health services are necessary to combat the fentanyl crisis. Legislative measures should focus on improving access to treatment for substance use disorders, expanding the availability of naloxone, and enhancing the surveillance of dark web marketplaces. International cooperation is also essential to disrupt the global supply chains responsible for the production and distribution of illicit fentanyl and its analogs [15].

Review limitations

While this review adheres to the PRISMA guidelines, certain limitations must be acknowledged. One of the key challenges is the potential for selection bias, as only studies published between 2010 and 2023 were included, which may have excluded relevant older research that could provide historical context on fentanyl use. Additionally, publication bias may have influenced the findings, as studies with statistically significant results are more likely to be published, potentially skewing the interpretation of fentanyl-related risks and trends.

Another limitation pertains to the heterogeneity of the included studies. Variations in sample sizes, study designs, and geographic focus could introduce inconsistencies in the interpretation of results. The lack of uniform data collection methods across different studies further complicates direct comparisons. To mitigate this, we have prioritized high-quality, peer-reviewed sources and highlighted discrepancies where applicable.

## Conclusions

The fentanyl epidemic among adolescents is a growing public health emergency that demands immediate and coordinated action. The complexity of this crisis lies in its multifactorial origins - spanning biological, psychological, social, and economic dimensions - and the unprecedented potency of the substance itself. Adolescents are particularly vulnerable due to their developmental stage, susceptibility to peer influence, and lack of awareness about the risks associated with synthetic opioids.

Addressing this crisis requires a combination of targeted prevention strategies, comprehensive education, community support, and policy reforms. Health professionals, educators, parents, and policymakers must collaborate to create a protective environment for adolescents, equipping them with the knowledge, skills, and resources needed to navigate the challenges of this critical life stage. Without such a concerted effort, the devastating toll of fentanyl on young lives will continue to rise, leaving a lasting impact on families, communities, and healthcare systems worldwide.
